# Subjective Cognitive Impairment and Mild Cognitive Impairment: risk factors in community-dwelling older adults

**DOI:** 10.1093/geroni/igaf122.2299

**Published:** 2025-12-31

**Authors:** Neyda Mendoza Ruvalcaba, Melina Rodríguez-Díaz, Karla Patricia Vazquez-Nuñez

**Affiliations:** Universidad de Guadalajara, Guadalajara, Jalisco, Mexico; Universidad de Guadalajara, Tonala, Jalisco, Mexico; Universidad de Guadalajara, Tonala, Jalisco, Mexico

## Abstract

**Introduction:**

Subjective cognitive impairment (SCD) and mild cognitive impairment (MCI) are preclinical states in the natural history of Major Neurocognitive Disorder or dementia. SCD has been studied as an indicator of MCI and future dementia, it is defined as persistent self-experienced decline in cognitive ability despite having normal performance on standardized tests.

**Objective:**

To identify the prevalence and risk factors for SCD and MCI in community-dwelling older adults from the Metropolitan Area in Guadalajara, Mexico.

**Method:**

Cross-sectional study, participated n = 1190 older adults (from 2018-2024). Mean age 71.44 years(SD = 7.5), education 9.24 years(SD = 4.8), 78% women(n = 927), 53% without a partner. After informed consent, trained gerontologists administered face-to-face interviews, measures of global cognitive functioning with the MMSE, meta-memory, and measurements of risk variables: sociodemographic data, physical and mental health. Data were analyzed with SPSSv28 and Odds Ratios (95% CI) were calculated.

**Results:**

Of the total participants 72.1% presented SCD, 11.6% MCI and 18.1% had normal cognitive functioning. The significant risk factors for SCD were age(OR = 1.31,CI=1.09-1.72,p=.025), sex(OR = 2.05, CI = 1.54 – 2.73, p < .001), education(OR = 1.21, CI = 1.03 – 1.53, p = .049), recent life crises(OR = 1.55, CI = 1.16 – 2.08, p = .002), loss of a loved one(OR = 1.32, IC = 1.02 – 1.74, p = .023), cancer(OR = 2.46 CI = 1.40 – 4.34, p < .001) and anxiety diagnosis(OR = 1.27, CI = 1.02 – 1.71, p = .049). The risk factors for MCI were presenting SCD(OR = 1.60, CI = 1.48 – 1.74, p < .001), having a previous diagnosis of depression(OR = 1.54, CI = 1.10 – 2.30, p = .016), while having no memory complaints was a protective factor(OR = .622, CI = .432-.895, p = .007).

**Conclusions:**

Detection of subjective cognitive impairment as an early manifestation of cognitive pathology is relevant to prevent or delay progression to MCI and dementia, allowing planning interventions to reverse/reduce the rate of conversion and its consequences on people’s lives and public health.

